# Systematic Review and Case Report on the Surgical Management of Pleomorphic Adenomas: Lessons on Recurrence and Error Prevention

**DOI:** 10.3390/jcm14134541

**Published:** 2025-06-26

**Authors:** Giulio Pagnani, Angela Palma, Fabrizio Bozza, Camilla Marsigli Rossi Lombardi, Roberto Becelli

**Affiliations:** 1 Maxillofacial Surgery Unit, Department of Odontostomatological and Maxillofacial Sciences, University of Rome “La Sapienza”, University Hospital Policlinico Umberto I, 00161 Rome, Italy; 2 Maxillofacial Surgery Unit, NESMOS Department, Faculty of Medicine and Psychology, S. Andrea Hospital, University of Rome “La Sapienza”, 00189 Rome, Italy; 3 Faculty of Medicine and Psychology, University of Rome “La Sapienza”, 00185 Rome, Italy

**Keywords:** pleomorphic adenoma, recurrence, parotidectomy, extracapsular dissection, parotid gland, head and neck surgery, surgery, complications, systematic review

## Abstract

**Background/Objectives:** Pleomorphic adenomas (PAs) are the most common salivary gland tumors, with a known risk of recurrence, especially after inadequate surgical excision. Understanding how surgical approach influences recurrence remains essential to optimize management. This study aimed to synthesize recurrence rates of PAs based on different surgical techniques and to illustrate the implications of surgical strategy through a representative case of multifocal deep lobe recurrence. **Methods:** A systematic review was conducted according to PRISMA 2020 guidelines. Three electronic databases (PubMed, Cochrane, and Google Scholar) were searched for studies published in the last ten years, reporting recurrence rates of PAs by surgical approach. Data were extracted on recurrence, complications, and tumor characteristics. Additionally, a complex clinical case of recurrent deep lobe PA (DLPA) was presented to contextualize the findings. **Results:** Fifteen studies were included, comprising a total of 2095 patients. Recurrence rates were 3.27% after extracapsular dissection (ED), 0.73% after partial superficial parotidectomy (PSP), and 2.41% after superficial parotidectomy (SP). Recurrent PA (RPA) is often multifocal and associated with increased risks of facial nerve palsy and positive surgical margins. The presented case involved five surgical procedures, with ultimate total parotidectomy and facial nerve preservation despite infiltrative recurrence in the prestyloid space. **Conclusions:** Techniques such as ED and PSP have demonstrated their efficacy and safety compared to more invasive approaches, although their application should be carefully evaluated based on tumor size and location. RPA remains a challenging entity to treat. Avoiding outdated techniques and ensuring evidence-based decision making may improve long-term outcomes in PA management.

## 1. Introduction

Pleomorphic adenomas, often termed benign mixed tumors, represent the most prevalent neoplasms of the salivary glands, particularly affecting the parotid gland [1]. These tumors comprise a mixture of epithelial and mesenchymal components, which contribute to their diverse clinical presentations [2]. Although classified as benign, pleomorphic adenomas can recur post-surgical excision, with recurrence rates documented to range from 1% to 45%, significantly influenced by surgical technique and the adequacy of tumor removal [3,4,5]. Effective surgical management necessitates striking a balance between complete tumor resection and the minimization of complications, such as facial nerve injury. However, the risk of recurrence remains a pressing concern, particularly in cases where surgical errors occur. Common pitfalls—including inadequate surgical margin clearance, capsular rupture, inappropriate technique selection, and failure to recognize critical anatomical structures—can lead to less favorable outcomes [6]. For instance, studies indicate that enucleation presents higher recurrence rates when compared to more extensive surgical interventions, such as superficial or total parotidectomy [7]. Additionally, thorough preoperative imaging and patient assessment, often overlooked, are vital in formulating effective surgical strategies [8,9].

This systematic review aims to identify prevalent surgical errors in managing pleomorphic adenomas of the parotid gland and their correlation with recurrence rates. In particular, a case report will be presented exemplifying the mismanagement of a pleomorphic adenoma, which our unit encountered during the treatment of the patient’s fourth recurrence, at a stage where the local disease had significantly advanced. By synthesizing the existing literature, this review seeks to emphasize the importance of meticulous surgical technique and strategic preoperative planning in reducing recurrence and improving patient outcomes. Ultimately, the goal is to provide evidence-based recommendations to enhance surgical practices and patient care.

## 2. Materials and Methods

The substructure of the systematic review is based on the PRISMA Statement. In conducting this systematic review, a comprehensive literature search was performed utilizing the MEDLINE (PubMed) database to identify relevant articles concerning the surgical management of pleomorphic adenomas of the parotid gland using the following syntax: (“Adenoma, Pleomorphic” [MeSH Terms]) AND (“Parotid Gland” [MeSH Terms]) AND (“Management” [MeSH Terms] OR “Errors” [MeSH Terms] OR “Quality of Health Care” [MeSH Terms]). Similarly, a search was conducted on Google Scholar using the syntax: “parotid gland” AND “pleomorphic adenoma” AND “surgical management” AND (“surgical errors” OR “management errors” OR “bad practice” OR “recurrence” OR “reoperation”), and on Cochrane Library using the terms: adenoma, pleomorphic AND parotid, gland.

Following this initial search, a series of automated filters were applied, restricting the results to studies published within the last ten years, in English, focusing on human subjects aged over 18 years, and containing full text. This limited timeframe is justified by the efforts to standardize parotid surgery nomenclature following the European Salivary Gland Society’s (ESGS) proposal in 2016 [10]. The subsequent adoption of unified classification, notably defining extracapsular dissection (ED), promotes more accurate and comparable surgical data, a trend supported by the increasing rate of citations to the ESGS’s classification paper over the last decade.

**Figure 1:**
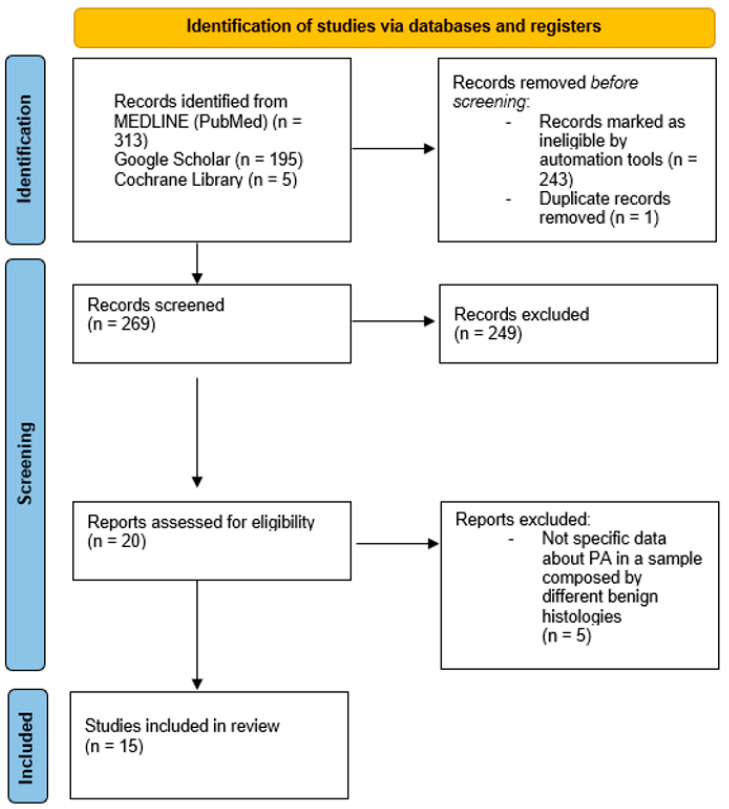
PRISMA flow diagram showing the process of identification and screening of articles reviewed [12].

Two authors conducted an independent screening of all published articles retrieved from the electronic databases. In the first phase of selection, the titles and abstracts were evaluated through predefined inclusion and exclusion criteria. The inclusion criteria encompassed cohort studies, case–control studies and studies on decision analytic models that specifically discussed surgical treatments and reported data on recurrence rates. Exclusion criteria eliminated studies that lacked robust data, focused solely on non-surgical management, or reported on populations outside the specified age group. In the second phase, full texts of the articles were reviewed by one author, and studies meeting the established inclusion and exclusion criteria were selected. Studies that did not report specific outcome data, particularly regarding recurrence rates in patients with pleomorphic adenomas, were excluded (Figure 1).

Data extraction was conducted to collect information on outcome measures such as the number of patients with pleomorphic adenoma (PA) included, recurrence rates and complications associated with various surgical techniques (defined as the percentage of patients experiencing recurrence or complications such as facial nerve paralysis (FNP), Frey’s syndrome, etc.). Additionally, data on resection margins (defined as the percentage of patients with positive margins), reoperation time (in years), preoperative assessments utilized (particularly the percentage of FNAC utilized and its accuracy), malignant transformation (presented as the percentage of patients who experience transformation in carcinoma ex pleomorphic adenoma), and the use of adjuvant therapies (percentage of patients who underwent adjuvant RT) were collected, where available. Statistical analyses of these studies were reported, including *p*-values, and focused on outcomes related to the comparison of surgical techniques or tumor location (superficial or deep lobe), the correlation between the number of reoperations and complications, and the use of FNAC concerning tumor location. Statistical significance was attributed to *p*-values less than 0.05 across all studies where analyses were presented. One of the included studies was a decision analytical model; in this case, sample data were not analyzed—there was no actual sample—but outcomes from the model were qualitatively collected and examined. Due to the significant heterogeneity among the studies in terms of patient populations, surgical techniques, and recorded complications, we found it useful to synthesize solely the data correlating recurrence with the surgical technique. Patients of the studies were categorized into three subgroups according to the technique used: extracapsular dissection, superficial parotidectomy (SP), and partial superficial parotidectomy (PSP). Studies solely comprising recurrent pleomorphic adenomas (RPAs) were excluded from this analysis, and only those reporting specific surgical techniques were included. In the included studies, patients diagnosed with primary PAs were selected, while those undergoing total parotidectomy were excluded. Each study’s outcomes were tabulated, resulting in average recurrence rates for each subgroup. Heterogeneity assessment among the groups involved Cochran’s Q test and I^2^ statistic, performed using Python (version 3.11.13) with the SciPy library (version 1.15.3) in Google Colab (Google LLC, Mountain View, CA, USA), followed by Exclusion Sensitivity Analyses to evaluate the robustness of the synthesized results. A continuity correction was applied to account for zero events. Cochran’s Q *p*-values were calculated using the chi-squared distribution. Statistical significance for heterogeneity was defined as *p* < 0.05. I^2^ values were interpreted as follows: 0–40%, heterogeneity might not be important; 30–60%, moderate heterogeneity; 50–90%, substantial heterogeneity; 75–100%, considerable heterogeneity. Statistical analyses were performed using Google. Two review authors independently assessed risk of bias using the risk of bias assessment tool for non-randomized studies of interventions (ROBINS-I V2) for non-randomized study. Any disagreement was resolved via discussion [11].

### Case Report

We report the case of a 42-year-old woman with a history of RPA of the right parotid gland, whose clinical course reflects the complexity and potential complications associated with the long-term management of this neoplasm.

**Figure 2:**
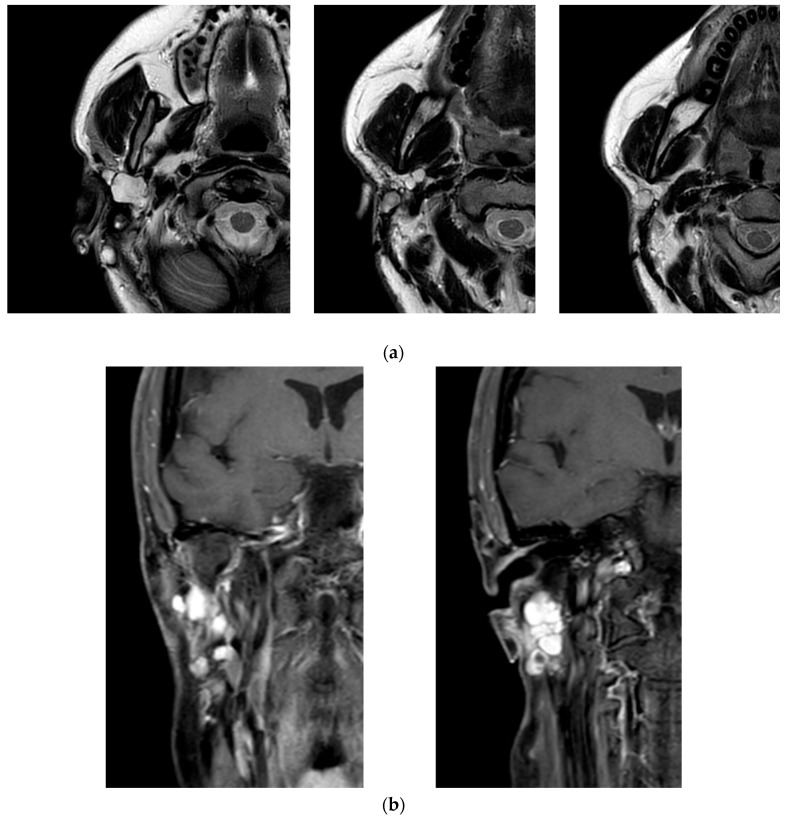
Magnetic resonance imaging (MRI) of the head and neck performed during the most recent recurrence of the pleomorphic adenoma. (**a**) Axial T2-weighted image showing a well-circumscribed, multilobulated lesion in the deep portion of the right parotid, with hyperintense signal and clear demarcation from adjacent structures. (**b**) Coronal contrast-enhanced T1-weighted image revealing intense peripheral enhancement of the lesion, consistent with a recurrent multinodular pleomorphic adenoma.

**Figure 3:**
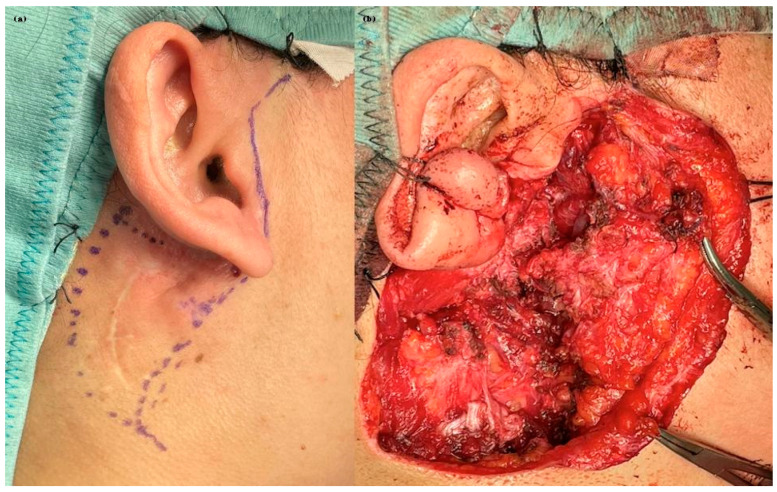
Intraoperative documentation from the most recent surgical procedure. (**a**) Preoperative skin marking illustrating the planned incision line and anatomical landmarks. (**b**) Intraoperative view following tumor excision, showing the exposed surgical bed after en bloc resection of the deep lobe and adjacent soft tissues, with preservation of the facial nerve trunk.

The patient’s clinical journey began nearly a decade ago, when she presented with a swelling in the right parotid region and subsequently underwent magnetic resonance imaging (MRI) of the neck, which revealed an oval, lobulated mass (22 × 15 mm) in the deep lobe of the right parotid gland, showing peripheral contrast enhancement. She initially underwent a superficial parotidectomy under general anesthesia at another institution. Over the following years, she underwent three additional surgical excisions under local anesthesia, consisting of the removal of retroauricular cutaneous and subcutaneous nodules. Notably, no FNAC or core biopsy was performed prior to these procedures. At the time of referral to our institution, histopathological confirmation of pleomorphic adenoma was already available. Given the characteristic radiological findings and multifocal recurrence pattern, no further preoperative cytological sampling was deemed necessary. A recent contrast-enhanced MRI revealed a well-capsulated, solid, multilobulated lesion in the deep right parotid bed, measuring 27 × 19 × 17 mm, located in the area of prior surgical interventions (Figure 2). The imaging findings were highly suggestive of yet another recurrence, prompting referral to our institution for further evaluation. The patient subsequently underwent a fifth surgical intervention—performed at our institution—consisting of total right parotidectomy with facial nerve preservation, combined with en bloc dissection of the parapharyngeal and submandibular spaces and excision of a laterocervical skin flap. Histopathological examination revealed foci of pleomorphic adenoma in continuity with resection margins, with tumor infiltration of skin, subcutaneous tissue, and skeletal muscle. One lymph node was removed and was negative for disease. Intraoperative photographs showing the preoperative surgical planning and the surgical field after tumor excision are provided in Figure 3. The postoperative course was uneventful, with satisfactory wound healing. However, the patient developed a right-sided facial nerve paralysis (FNP) (House–Brackmann grade V). Histopathological examination confirmed the presence of tumor foci in continuity with the resection margins, reflecting the infiltrative and multifocal nature of the disease. The surgical procedure was carried out as extensively as possible, with wide excision of the deep lobe and adjacent soft tissues. The patient is currently being evaluated for adjuvant radiotherapy. A follow-up MRI is scheduled at 6 months. While long-term surveillance remains essential, the combined surgical and oncological approach is expected to significantly reduce the risk of further recurrence. This case underscores the challenging nature of managing recurrent pleomorphic adenomas, particularly when the disease progresses over a long period through multiple surgical interventions. It highlights the importance of comprehensive preoperative planning, accurate anatomical evaluation, and specialist surgical expertise from the earliest stages to minimize the risk of recurrence and procedural morbidity.

## 3. Results

### 3.1. Study Selection

The search strings employed were specifically designed to capture studies related to pleomorphic adenomas, parotid gland management, and recurrence, resulting in a total of 513 articles, of which 313 were obtained from the Medline database, 195 from Google Scholar, and 5 from the Cochrane Library. The automated filtering process ultimately yielded 270 articles, indicative of the growing body of literature surrounding this topic. One of these 270 articles was excluded because it was a duplicate present in two databases. Of these, 249 studies were discarded because, after reviewing titles and abstracts, it appeared that these papers clearly did not meet the criteria, further refining the selection to 20 pertinent articles. The full text of the remaining 20 citations was examined in more detail. At the end of the screening process, 15 studies were definitively included in the review.

### 3.2. Study Characteristics

Characteristics of the included studies have been summarized in Table 1. The study designs included were the following: 12 retrospective studies, 1 prospective study, 1 case-control study, and 1 analytical model development. The studied populations consisted of patients diagnosed with primary PAs, while others focus on RPAs, and some include both (Table 1). In total, 2095 cases of pleomorphic adenoma were analyzed.

Of the 15 studies included in this review, 7 (47%) directly employed the ESGS classification for parotidectomies, enhancing data comparability. While the remaining 8 (53%) did not explicitly state adherence to the ESGS guidelines, their surgical technique descriptions were reconcilable with the ESGS categories. Notably, the surgical technique described as partial superficial PSP in these articles consistently aligns with Parotidectomy I or Parotidectomy II according to the ESGS classification.

### 3.3. Risk of Bias in Studies

**Figure 4:**
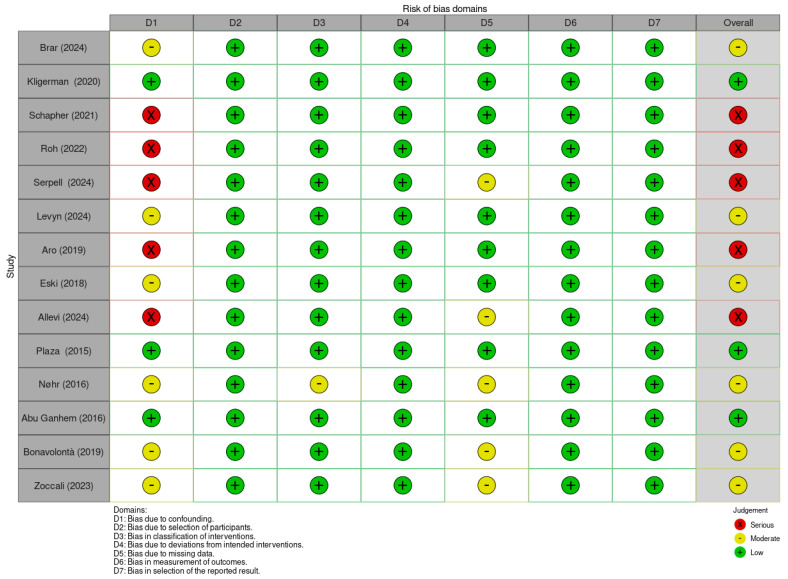
Summary of risk of bias assessment for non-randomized controlled trials using ROBINS-I V2 tool. Brar et al. (2024) [13]; Kligerman et al. (2020) [14]; Schapher et al. (2021) [15]; Plaza et al. (2015) [16]; Eski et al. (2018) [17]; Allevi et al. (2024) [18]; Levyn et al. (2024) [19]; Aro et al. (2021) [20]; Nøhr et al. (2016) [21]; Roh (2022) [22]; Roh (2025) [23]; Serpell et al. (2024) [24]; Abu Ghanem et al. (2016) [25]; Bonavolontà et al. (2019) [26]; Zoccali et al. (2019) [27].

The ROBINS-I V2 (Risk Of Bias In Non-randomized Studies of Interventions, Version 2) is a comprehensive tool designed to evaluate risk of bias for each specific result within an individual study [11]. This tool assesses seven domains through which bias might be introduced: bias due to confounding, bias in the selection of participants into the study, bias in classification of interventions, bias due to deviations from intended interventions, bias due to missing data, bias in the measurement of outcomes, and bias in the selection of the reported result. The judgments within each domain carry forward to an overall risk of bias of: “Low risk of bias”, “Moderate risk of bias”, “Serious risk of bias”, “Critical risk of bias”, or “No information”. In this review, articles were primarily assessed based on quantitative outcomes related to tumor recurrence, which is a direct and objective measure. When such quantitative data were absent or expressed qualitatively, the focus shifted to outcomes potentially associated with recurrence, such as capsular rupture or tumor spillage, which are considered indirect indicators. In cases where these were not available, the evaluation concentrated on surgical complications like facial nerve paralysis, which, while indirect, impact patient morbidity. The article by Roh 2024 was excluded by this evaluation, due to the lack of relevant quantitative recurrence data. Overall, the studies showed moderate to high risk of bias, mainly due to confounding factors, making the overall bias assessment critical for interpreting the evidence (Figure 4).

### 3.4. Limitations

This review acknowledges several limitations. The reliance on primarily retrospective studies introduces potential selection bias, and while statistical tests suggest low heterogeneity, clinical differences in surgical techniques and patient populations across studies could exist. Data availability varied, with some studies lacking detailed information on resection margins or complications. Furthermore, the limited follow-up duration in some studies may underestimate true recurrence rates, potentially affecting the generalizability of the findings. Publication bias, where studies with statistically significant results are more likely to be published, could also skew the synthesized results. Future research should prioritize prospective designs and standardized data collection to enhance the certainty of evidence in this area.

### 3.5. Results of Individual Studies

#### 3.5.1. Study 1

Multiple recurrent PAs were more likely to require at least partial nerve sacrifice at the time of reoperation (*p* = 0.0092). Significantly worse long-term FN outcomes were seen following surgery for multiple recurrent PA (*p* = 0.008). There was no significant difference between the rate of re-recurrence following first revision surgery vs. second-fourth revision surgery. Time to reoperation was significantly shorter between the first and second revision surgery than between the primary surgery and first revision (*p* = 0.0017) [13].

#### 3.5.2. Study 2

A decision analytical model was constructed to compare upfront elective parotidectomy with observation in patients 50 years or older. The age thresholds at which observation became more beneficial than parotidectomy were 88.5 years for patients with superficial lobe tumors and 83.4 years for patients with deep lobe tumors). There was no significant difference in outcomes between parotidectomy and observation among patients aged 70 to 80 years [14].

#### 3.5.3. Study 3

ED was performed in 29.7%, and other surgical modalities (OSMs), including facial nerve dissection, in 70.3%. After the long-term follow-up, 98% of all the patients (n = 96/98) were recurrence free. No significant differences were noted when recurrence rates were compared (*p* < 0.331). ED resulted in significantly lower FNP rates compared to OSMs (*p* < 0.001). FNP rates significantly increased with size and location of the tumors according to ESGS categories (temporary and permanent FNP, *p* = 0.04). Surgical invasiveness corresponded to a significant increase in the incidence of Frey’s syndrome (*p* < 0.001) [15].

#### 3.5.4. Study 4

PSP resulted in fewer early and late complications than SP, with similar recurrence rates. Temporary FNP was found in 4% of PSP, vs. 12% of SP, a significant difference. Sialocele was more common after PSP (28% vs. 16%), a significant difference. No recurrences have been observed during a minimum follow-up of 4 years (median = 6 years, range = 4–8 years) [16].

#### 3.5.5. Study 5

No significant differences were found between the group that underwent SP and the group that underwent PSP in terms of tumor size, complications, or recurrence rates [17].

#### 3.5.6. Study 6

This study reported the following incidences of complications following ED: temporary facial nerve dysfunction in 6.70% of patients; permanent facial nerve dysfunction in 0% of patients; Frey’s syndrome manifestations were 0.52%. Five patients (2.58%) experienced a recurrence: three were pleomorphic adenomas, and two were Warthin’s tumors. This case series shows how a standardized approach to extracapsular dissection for PA tumors yields favorable results [18].

#### 3.5.7. Study 7

While the sensitivity of FNA for diagnosing PA was higher in SLPA (90.0%), it remained respectable at 80% for DLPA. Postsurgical complications were more frequent in DLPA (41% vs. 30% in SLPA, *p* = 0.025). The most frequent sequela in both groups was facial nerve weakness, which was observed in higher rates in DLPA patients (33% vs. 22% in SLPA, *p* = 0.01). The rate of Frey’s syndrome was higher after DLPA resections compared to superficial lobe tumor, although this difference was not statistically significant. DLPA also showed higher recurrence rates (*p* = 0.016) [19].

#### 3.5.8. Study 8

This study was a retrospective review of patients with multiple RPAs. Primary tumors were not multifocal, while at the first recurrence, multifocal disease was found in 59% of cases, positive surgical margins were found in 41%. At the second recurrence, rupture of the tumor capsule and direct spillage of tumor cells was reported in 29% of cases, positive surgical margins in 14%, and multifocal disease in 57%. At the third recurrence, multifocal tumors were found in 71% of cases. The time interval between recurrences shortened after each recurrent event, with a median of 5.8 years between the first and the second recurrence [20].

#### 3.5.9. Study 9

Statistically significant associations were found between the number of surgeries and permanent facial nerve dysfunction of all degrees (*p* = 0.001) in patients with RPA. This means that for every additional surgery, the risk of any degree of FNP increased by a factor of 1.43. Risk of different degrees of paresis after the second–fourth surgeries was found (*p* < 0.05). The risk of FNP increases linearly with each additional surgery [21].

#### 3.5.10. Study 10

In this study, 248 patients underwent PSP via periauricular incision. Median operation time was 55 min. No patients had tumor capsular disruption during excision. Temporary numbness of the affected auricular region and temporary FNP were found in 31.5% and 5.6% of patients. Patients’ satisfactions for incision scar and facial deformity were very high with median VAS scores of 9 and 10. The postoperative secretory function of the affected gland was similar to that of the unaffected gland. No patients had recurrences [22].

#### 3.5.11. Study 11

Six patients with PA underwent ECD via the preauricular approach. The median duration of the procedure was 55 min, with an average blood loss of 25 mL. No facial nerve or Stensen’s duct injury, or tumor capsule ruptures were recorded. Patients reported high satisfaction regarding the appearance of the incision scar and facial contour. The postoperative secretory function of the affected gland closely mirrored that of the unaffected gland. No cases of recurrence were observed [23].

#### 3.5.12. Study 12

Histological margin involvement was similar in central and peripheral tumors, both overall and for superficial and DLPA, but was more common in central DLPA (*p* = 0.003). DLPA and total parotidectomy were associated with FNP (*p* = 0.01). Facial nerve monitoring reduced the risk of palsy (*p* < 0.01) [24].

#### 3.5.13. Study 13

The study was a retrospective review of 22 patients operated on for recurrent PA. The mean interval between recurrences was 7 years (first recurrence) and 6 years (second). A younger age at initial treatment was associated with a second recurrence. Facial nerve paralysis was found in only 9% of patients after surgery for recurrence. There were no cases of malignant transformation. Adjuvant radiotherapy was given to nine patients [25].

#### 3.5.14. Study 14

Hematoma and hypoesthesia occurred significantly more frequently following SP than ED (8.9% compared to 7.7%, and 16.8% compared to 5.6%, respectively). Transient facial nerve injury, Frey syndrome, and FNP were also significantly more common after SP than ED (23.6% versus 1.5%, 6.7% versus 1%, and 6.7% versus 0%, respectively). The ED group experienced more recurrences than the SP group, although this difference was not statistically significant [26].

#### 3.5.15. Study 15

Transient facial nerve palsy, FNP, and Frey syndrome were significantly more frequent after SP than after ED (*p* < 0.001). The recurrence rate was significantly more frequent after ED rather than when performing SP (70% vs. 30% [*p* < 0.001]) [27].

### 3.6. Results of Synthesis

In synthesizing the results for surgical techniques, we filtered and divided the studies into three subgroups (ED, PSP, SP), thereby reducing biases related to methodologies and populations. In total, 8 of the 15 studies were included in this synthesis. The statistical synthesis showed that ED had a low recurrence rate of approximately 3.27%, indicating its effectiveness, while the overall recurrence rate for PSP was about 0.73%, and SP reported 2.41%. The Cochran’s Q Test and I^2^ statistic showed no significant heterogeneity within these subgroups. For the ED group Q = 0.0563, I^2^ < 40%, *p* = 0.9984. PSP yielded Q = 0.0692, I^2^ < 40%, *p* = 0.9953. Similarly, SP showed Q = 0.0467, I^2^ < 40%, *p* = 0.9974. The absence of statistically significant heterogeneity (*p* > 0.05, I^2^ < 40%, in all groups) suggests consistency across studies for each technique. The sensitivity analysis for the PSP subgroup revealed variability in recurrence rates based on the study that was excluded. Excluding Roh (2025) [23] resulted in a higher recurrence rate of 1.84%, while excluding Serpell led to a complete absence of recurrences. The sensitivity analysis for the SP subgroup shows that excluding Bonavolontà (2019) [26] results in a complete absence of recurrence. For the ED subgroup, the lowest rate (2.45%) was obtained after excluding Bonavolontà (2019) [26], while the highest rate (3.54%) came after excluding Schapher (2021) [15]. The similarity of these results indicates robustness in the findings. Potential biases due to missing results may arise from the follow-up period, which is at a minimum of 12 months for most studies (except for Plaza and Roh 2025) [16,23]. This limited follow-up duration may have led to an underestimation of recurrence rates, emphasizing the need for further research to enhance the certainty of evidence in this area.

## 4. Discussion

### 4.1. Preoperative Assessment

Accurate preoperative assessment is paramount for effective surgical planning. Fine-needle aspiration cytology (FNAC) plays a vital role in this process, although its sensitivity varies depending on tumor location and the potential for sampling error. Levyn et al. (2024) [19] provide a comparative analysis of FNAC sensitivity for SLPAs and DLPAs, emphasizing the utility of CT-guided biopsies for DLPAs, where FNAC alone may be less reliable. They found FNAC sensitivity being 90% for SLPA but remaining respectable at 80% for DLPA [19]. Aro et al. (2021), in their study of recurrent PAs, discuss the accuracy of FNAC in diagnosing carcinoma ex pleomorphic adenoma (CXPA), noting that FNAC correctly indicated malignancy in only two of three CXPAs [11]. Kligerman et al. (2020), in their decision analytical model, underscore the importance of preoperative diagnosis in treatment planning, especially for elderly patients where observation may be a viable alternative [14].

### 4.2. Surgical Techniques for Primary PA

The choice of surgical technique for primary PAs aims to balance complete tumor removal with minimizing morbidity. The literature provides substantial evidence that transitioning from enucleation to SP has significantly decreased the recurrence rates of pleomorphic adenomas [28]. Additionally, as ED gains popularity, past reports have indicated that the risk of tumor recurrence following ED may not be greater than that associated with more extensive surgical approaches [29,30,31]. Other techniques, such PSP, which involves resecting at least one segment of the parotid gland with exposure of the facial nerve, are increasingly recognized. Eski et al. (2018) reported no significant differences between SP and PSP in tumor size, complications, or recurrence rates [17], while Plaza et al. (2015) found that PSP has less facial paresis than SP [16]. Schapher et al. (2021) compare ED with other surgical modalities (OSMs; e.g., partial superficial parotidectomy, superficial parotidectomy or complete parotidectomy), in their retrospective analysis emphasizing that ED is associated with lower FNP rates compared to OSMs, while no significant differences were noted when recurrence rates were compared; moreover, they highlight the role of the ESGS classification in choosing the surgical technique, with more extensive techniques used for higher ESGS categories, reflecting the increasing necessity to dissect the main trunk of the facial nerve [15] (Table 2, Table 3, Table 4 and Table 5). However, in contrast to the previously cited literature, Zoccali et al. (2023) found a higher recurrence rate for ED [27]. Conversely, this is not as true for another Italian study conducted a few years earlier by Bonavolontà et al. (2019) [26].

### 4.3. Management of the Deep Lobe

Deep lobe PAs (DLPAs) present unique surgical challenges due to their anatomical relationship to the facial nerve and surrounding structures.

Levyn et al. (2024) report higher complication and recurrence rates for DLPA, primarily due to difficulties in achieving clear margins during excision [19]. Serpell et al. (2024) suggest to approach DLPAs by resection of part of the superficial lobe to expose the relevant facial nerve branches and then to excise the deep lobe tumor (near total parotidectomy). They found that deep lobe tumors were associated with facial nerve palsy [24]. Kligerman et al. (2020) consider tumor location (deep vs. superficial lobe) when determining the age threshold for observation. Observation became more beneficial than parotidectomy at the age of 88.5 years for patients with superficial lobe tumors and 83.4 years for patients with deep lobe tumors. This temporal difference is due to the study attributing a higher risk of facial nerve paralysis to total parotidectomy for benign deep lobe tumors [14].

### 4.4. PA of the Parapharyngeal Space

PAs from the deep lobe of the parotid gland can extend into the parapharyngeal space (PPS), and recurrent PAs of the parotid may invade this area, as noted by Aro et al. [20]. Alternatively, PAs may originate de novo from minor salivary glands or ectopic tissue within the PPS [32]. Traditionally, the management of PPS tumors has relied on transcervical or transcervical–transparotid surgical approaches. However, recent surgical advancements offer new options, such as transoral robotic surgery (TORS), as well as transoral and robotic-assisted transcervical–retroauricular methods. These techniques show promise for PPS tumor resection, though their exact indications and limitations are not fully defined [33,34,35]. TORS, in particular, has demonstrated potential for managing parapharyngeal PAs, despite studies indicating a capsular rupture rate of 10–15% [33,36]. Regardless of the approach, it remains crucial to adhere to the principle of extracapsular dissection for deep lobe PAs, ensuring a thin layer of normal salivary tissue is left attached to the tumor specimen to reduce recurrence risks.

### 4.5. Surgical Techniques for Recurrences

Managing recurrent PAs requires careful consideration of a surgical approach. Brar et al. (2024) [13] performed a retrospective review of patients undergoing reoperation for recurrent PAs, revealing that multiple recurrences substantially increase the risk of facial nerve sacrifice. Their study indicated that facial nerve monitoring had limited benefits in such recurrent cases, suggesting that the recurrent surgical landscape poses challenges that differ from those in primary cases [13]. Aro et al. (2021) [20] investigated patients with repeatedly recurring PAs, noting that the surgical management frequently included resection of multifocal recurrence, which complicates further interventions. Their findings revealed that as recurrences accumulated, they tended to be multifocal, necessitating a shift in surgical techniques [20]. Abu Ghanem et al. (2016) report enucleation, superficial parotidectomy, and subtotal parotidectomy for recurrences, indicating that the choice of technique depends on the extent and location of the recurrent tumor [25].

### 4.6. Surgical Margins

While complete tumor removal is essential, the extent of surrounding normal tissue resection is a key consideration. Serpell et al. (2024) found that most tumors were peripherally situated, but peripheral location was not associated with decreased margin involvement, except for central deep lobe tumors. However, there was a significant higher involvement in deep lobe central tumors compared to superficial lobe central tumors [24]. Aro et al. (2021) found positive surgical margins in 41% of first recurrences, highlighting the challenge of achieving clear margins in revision surgeries [20]. In the Brar et al. study, when comparing outcomes after surgery for singly recurrent and multiple recurrent tumors in their group, there was no significant difference in the incidence of positive margins on pathology or in the likelihood of finding multicentric disease [13,37].

### 4.7. Multifocality

Multifocality complicates surgery for recurrence, as noted by Brar et al. (2024) [13] and Aro et al. (2021) [20] who state that multifocality increases with recurrences. In particular, in the latter among patients with recurrent adenomas, 59% of tumors at first recurrence were multifocal, while 71% were multifocal at third recurrence. Abu Ghanem et al. (2016) acknowledge multifocality as a characteristic of recurrent PAs, with multifocal recurrence observed on histologic analysis in 15 of 22 patients (68%) [25]. This highlights that the awareness of potential multifocality is crucial for surgical planning, as inadequate recognition can lead to incomplete resection and increased rates of further recurrences.

### 4.8. Reoperation Time

Brar et al. (2024) [13] detailed how the interval between reoperations tends to decrease with each successive recurrence. They reported that the time to reoperation between the first and second revision surgeries was significantly shorter than that between primary surgery and the first revision, with an average decrease noted in timing for repeated interventions [13]. This phenomenon may stem from underlying tumor biology and the cumulative effects of previous surgical interventions, which can result in more aggressive disease behavior over time or that patients are more vigilant in seeking treatment. Aro et al. report that the time to the first reoperation is significantly longer than the time between subsequent reoperations, with a median of 5.8 years between the first and second recurrence [20]. Abu Ghanem et al. (2016) [25] also reflected on reoperation timelines in their smaller cohort, noting that younger patients at initial treatment often experienced more rapid recurrence intervals. Their findings suggest that the age of presentation may determine both the frequency of recurrences and the urgency of subsequent surgical interventions [25].

### 4.9. Facial Nerve Injuries During Revision Surgeries

FNP is a significant concern during revision surgeries. Brar et al. (2024) [13] find that multiple recurrences increase the risk of facial nerve sacrifice and worsen long-term outcomes. Additionally, patients who received intraoperative facial nerve monitoring had no significant difference in postoperative facial nerve function compared to those who did not, outlining that facial nerve monitoring may yield limited benefits in such cases [13]. Nøhr et al. (2016) [21] find a statistically significant association between the number of surgeries and FNP. Their study suggested a linear increase in the risk of nerve dysfunction with each additional surgical intervention [21]. In contrast, Abu Ghanem et al. (2016) in their small cohort of recurrent PA, reported no facial nerve paralysis after the first surgery for recurrence [25].

### 4.10. Other Complications

Beyond facial nerve injuries, other complications can occur. Schapher et al. (2021) reported complications such as Frey’s syndrome and temporary facial nerve dysfunction, highlighting that the incidence of complications was significantly higher in patients undergoing more extensive surgical procedures compared to those treated with less invasive techniques such as ED, which displayed substantially lower rates of Frey’s syndrome [15]. Similarly, Bonavoltontà et al. (2019) [26] and Zoccali et al. (2023) [27] concluded that transient facial nerve palsy, FNP, and Frey syndrome were significantly more frequent after SP than after ED. On the other hand, Plaza et al. (2015) find that PSP has more sialocele than SP [16]. Levyn et al. (2024) provided similar insights regarding the complications encountered in patients with deep lobe tumors, noting a 41% complication rate that necessitated ongoing management and vigilance [19].

### 4.11. Adjuvant Radiotherapy

The role of adjuvant radiotherapy in the management of pleomorphic adenomas, particularly for recurrent cases, has garnered attention in the recent literature. Pleomorphic adenoma (PA) is a slow-growing benign tumor, raising questions about the effectiveness of radiotherapy as a treatment option. This presents a challenging dilemma, especially considering that many patients affected are young and thus are at risk for the full spectrum of radiation-related toxicities (dermal changes, xerostomia, hypothyroidism, and accelerated arteriosclerosis). Nonetheless, a significant concern associated with adjuvant radiotherapy is the potential development of secondary malignancies, which may arise 20–30 years post-treatment. While the risk of radiation-induced malignant transformation in benign parotid tumors has been frequently noted, only a limited number of studies have successfully demonstrated the actual induction of these secondary cancers [38,39,40,41]. In the studies included in this review, there is a lack of data to evaluate its benefit statistically.

### 4.12. Carcinoma Ex Pleomorphic Adenoma

The potential for malignant transformation, specifically to carcinoma ex pleomorphic adenoma (CXPA), is a critical consideration in managing PAs, particularly in recurrent cases. While some studies, like Brar et al. (2024), did not observe malignant transformation [13], others, such as Aro et al., reported a 6% rate of CXPA in their series of recurrent tumors [20]. These findings underscore the importance of long-term follow-up and surveillance, even after seemingly successful surgical management, to detect any signs of malignant change.

## 5. Conclusions

Techniques such as ED and PSP have demonstrated their efficacy and safety compared to more invasive approaches, although their application should be carefully evaluated based on tumor size and location. RPA remains a challenging entity to treat, as it is often multifocal and associated with increased risks of facial nerve palsy and positive surgical margins. To our knowledge, this is the first study to integrate a systematic synthesis of recurrence rates across surgical approaches for parotid PA. This combined perspective links population-level evidence with real-world surgical decisions, highlighting that awareness of errors, adherence to evidence-based practices, and ongoing re-evaluation are crucial for optimizing patient outcomes in PA management [42]. 

The evidence included in this review is limited by the observational and main retrospective nature of these studies and sample sizes, which may affect the generalizability of the findings. 

## Figures and Tables

**Table 1:** List of included studies and their main characteristics.

N	Study	Sample	Subjects	Design	Main Finding
**1**	**Brar et al. (2024)** [13]	Patients with recurrent RPAs	38	Retrospective study	Time to reoperation was significantly shorter between the first and second revision surgery than between the primary surgery and first revision; no significant difference between the rate of re-recurrence following first revision surgery vs second-fourth revision surgery.
**2**	**Kligerman et al. (2020)** [14]	≥50 years old patients with PAs	/	Decision analytical model	Observation may be more advantageous than surgical intervention in older patients.
**3**	**Schapher et al. (2021)** [15]	Patients with PAs	182	Retrospective study	ED compared to SP does not show significant differences in recurrence rates while show lower FNP rates.
**4**	**Plaza et al. (2015)** [16]	Patients with BPT	50	Case-control retrospective study	PSP resulted in fewer complications than SP, with similar recurrence rates.
**5**	**Eski et al. (2018)** [17]	Patients with superficial BPT	39	Retrospective study	Segmental superficial parotidectomy is a safe and effective option.
**6**	**Allevi et al. (2024)** [18]	Patients with BPT	194	Retrospective study	ED yields favorable results with low complication and recurrence rates.
**7**	**Levyn et al. (2024)** [19]	Patients with SLPAs and DLPAs	369	Retrospective study	DLPAs have higher complication and recurrence rates than SLPAs.
**8**	**Aro et al. (2021)** [20]	Patients with RPAs	47	Retrospective study	Multiple recurrences shorten time intervals and increase multifocality.
**9**	**Nøhr et al. (2016)** [21]	Patients with RPAs	198	Retrospective study	The risk of paresis increases linearly with each additional surgery.
**10**	**Roh (2022)** [22]	Patients with SLPAs	248	Retrospective study	Demonstrated safety and feasibility of using a preauricular incision for PSP.
**11**	**Roh (2025)** [23]	Patients with SLPAs	8	Retrospective study	Demonstrated safety and feasibility of using a preauricular incision for ED.
**12**	**Serpell et al. (2024)** [24]	Patients with superficial BPT	303	Prospective study	PSP is a safe and adequate technique for benign parotid tumor
**13**	**Abu Ghanem et al. (2016)** [25]	Patients with recurrent PAs	22	Retrospective study	Time to reoperation shorter between first and second revision surgery compared to primary surgery and first revision. Multiple recurrences associated with higher risk of FNP.
**14**	**Bonavolontà et al. (2019)** [26]	Patient with PA	297	Retrospective study	ED compared to SP do not show significant differences in recurrence rates while show lower morbidity rates.
**15**	**Zoccali et al. (2019)** [27]	Patient with PA	255	Retrospective study	Recurrence rate was significantly more frequent after ED than SP, while morbidity was lower in ED than SP.

BPT: benign parotid tumor; DLPA: deep lobe pleomorphic adenoma; ED: extracapsular dissection; FNP: facial nerve paralysis; PA: pleomorphic adenoma; PSP: partial superficial parotidectomy; RPA: recurrent pleomorphic adenoma; SLPA: superficial lobe pleomorphic adenoma.

**Table 2:** ED recurrence rate across studies.

Study	Patients	Recurrences
**Schapher (2021)** [15]	31	0
**Roh (2025)** [23]	8	0
**Allevi (2024)** [18]	165	5 (3.03%)
**Bonavolontà (2019)** [26]	194	8 (4.1%)
**All Studies**	**398**	**13 (3.27%)**

**Table 3:** PSP recurrence rate across studies.

Study	Patients	Recurrences
**Roh (2022)** [22]	248	0
**Serpell (2024)** [24]	121	3 (2.48%)
**Eski (2018)** [17]	17	0
**Plaza (2015)** [16]	25	0
**All Studies**	**411**	**3 (0.73%)**

**Table 4:** SP recurrence rate across studies.

Study	Patients	Recurrences
**Schapher (2021)** [15]	30	0
**Eski (2018)** [17]	22	0
**Plaza (2015)** [16]	25	0
**Bonavolontà (2019)** [26]	89	4 (4.49%)
**All Studies**	**166**	**4 (2.41%)**

**Table 5:** Proposed classification for benign tumors of the parotid gland, organized by size and localization according to the European Salivary Gland Society (ESGS) guidelines.

Category	Size Localization
I	Tumor < 3 cm, superficial (outer surface), mobile and close to parotid borders
II	Tumor < 3 cm, deep, or far from parotid borders
III	Tumor > 3 cm involving two levels
IV	Tumor > 3 cm involving more than two levels

## Data Availability

Due to the limited amount of extracted and processed data, and considering that this is primarily a qualitative systematic review, none of the following materials are publicly available: template data collection forms, data extracted from included studies, data used for analyses, analytic code, or any other materials used in the review.

## Abbreviations

The following abbreviations are used in this manuscript:

BPT	Benign parotid tumor
CXPA	Carcinoma ex pleomorphic adenoma
DLPA	Deep lobe pleomorphic adenoma
ED	Extracapsular dissection
FNAC	Fine needle aspiration cytology
FNP	Facial nerve paralysis
MRI	Magnetic resonance imaging
OSM	Other surgical modalities
PA	Pleomorphic adenoma
PPS	Parapharyngeal space
PSP	Partial superficial parotidectomy
RPA	Recurrent pleomorphic adenoma
SLPA	Superficial lobe pleomorphic adenoma
TORS	Transoral robotic surgery
